# Splenic Blood Flow Increases after Hypothermic Stimulus (Cold Pressor Test): A Perfusion Magnetic Resonance Study

**DOI:** 10.1155/2019/8437927

**Published:** 2019-05-23

**Authors:** Nicola Galea, Giulia Cundari, Cristian Borrazzo, Giacomo Pambianchi, Angelica Bracci, Edoardo Rosato, Marco Francone, Iacopo Carbone, Carlo Catalano

**Affiliations:** ^1^Department of Radiological, Oncological and Pathological Sciences, Sapienza University of Rome, Rome 00161, Italy; ^2^Department of Experimental Medicine, Sapienza University of Rome, Rome 00161, Italy; ^3^Medical Physics Unit, Policlinico Umberto I, Rome 00161, Italy; ^4^Department of Clinical Medicine, Clinical Immunology Unit-Scleroderma Center, Sapienza University of Rome, Rome 00161, Italy

## Abstract

The Cold Pressor Test (CPT) is a novel diagnostic strategy to noninvasively assess the myocardial microvascular endothelial-dependent function using perfusion magnetic resonance imaging (MRI). Spleen perfusion is modulated by a complex combination of several mechanisms involving the autonomic nervous system and vasoactive mediators release. In this context, the effects of cold temperature on splenic blood flow (SBF) still need to be clarified. Ten healthy subjects were studied by MRI. MRI protocol included the acquisition of GRE T1-weighted sequence (“first pass perfusion”) during gadolinium administration (0.1mmol/kg of Gd-DOTA at flow of 3.0 ml/s), at rest and after CPT. Myocardial blood flow (MBF) and SBF were measured by applying Fermi function deconvolution, using the blood pool input function sampled from the left ventricle cavity. MBF and SBF values after performing CPT were significantly higher than rest values (SBF at rest: 0.65 ± 0.15 ml/min/g Vs. SBF after CPT: 0.90 ± 0.14 ml/min/g, p: <0.001; MBF at rest: 0.90 ± 0.068 ml/min/g Vs. MBF after CPT: 1.22 ± 0.098 ml/min/g, p<0.005). Both SBF and MBF increased in all patients during the CPT. In particular, the CPT-induced increase was 43% ± 29% for SBF and 36.5% ± 17% for MBF. CPT increases SBF in normal subjects. The characterization of a standard perfusion response to cold might allow the use of the spleen as reference marker for the adequacy of cold stimulation during myocardial perfusion MRI.

## 1. Introduction

In humans, splenic vascularization is modulated by several mechanisms, among which the predominant role is played by the autonomic nervous system (ANS), through both neural and humoral factors [1].

Splenic sympathetic innervation originates from the thoracic spinal cord (T3-T12) and ends diffusely in the spleen capsule and periarteriolar regions [1, 2] through the celiac-mesenteric plexus and the splenic neurovascular bundle.

ANS-mediated vasomotility relies on the contraction of smooth muscle cells as the result of the stimulation of *α*1- and *β*-adrenergic receptors (AR) by norepinephrine (NE), whereas sympathetic stimulation acts on splenic blood flow (SBF) also modulating spleen volume [1, 3, 4]. In addition, the activation of adenosine A1-receptors causes vasoconstriction in spleen vessels, as demonstrated by the physiological phenomenon of adenosine-induced “splenic switch-off” during stress-perfusion cardiac magnetic resonance [5, 6]. Finally, as regards the parasympathetic innervation, some anatomical studies demonstrated the low presence of cholinergic nervous fibres in the spleen [7], and it is known that the spleen contains acetylcholine (Ach) [8], which is probably partially produced and released by CD4+ T-Cells [9]; however, the role of parasympathetic nervous fibres in splenic vasomotion is still a matter of debate [7].

Despite the different mechanisms involved in spleen vasomotion having been largely investigated by several studies [1, 3, 4], the combination of their effects in response to specific stimuli (physical, physiological, or pharmacological) still needs to be clarified.

Recently, the assessment of myocardial perfusion reaction to hypothermic stimulus, using magnetic resonance imaging (MRI), has been proposed as an innovative and noninvasive strategy to investigate the coronary microvascular function [10–12]. This technique consists of evoking the endothelium-dependent vasodilation induced by the adrenergic stimulation (sympathetic activation and catecholamine release by adrenal medulla) after submerging both hands in ice water for four minutes (Cold Pressor Test: CPT).

In this study, our purpose was to assess the modification of splenic perfusion in response to CPT in normal subjects. The identification of a standard response of SBF after the CPT could emerge as an adequacy marker of the correct execution of cardiac perfusion MRI using cold stimulus.

## 2. Materials and Methods

### 2.1. Patients

We studied a cohort of 10 healthy, nonsmoking subjects (five men and five women, mean age 47.3 ± 7.3) with no generic contraindications to MRI or gadolinium-based contrast agent administration (e.g., pregnancy, chronic renal failure, and history of hypersensitivity to contrast agent), no hypertension, no blood dyscrasias or disorders, no portal hypertension, and no recent infectious diseases.

Exclusion criteria included abnormalities in splenic morphology or dimensions and splenic focal lesions; in this perspective, all subjects were submitted to splenic ultrasound prior to enrolment. The local ethics committee approved the study; the informed consensus was obtained for each subject.

### 2.2. MRI Protocol

All MRI exams were performed on a 1.5 T scanner (Avanto Magnetom, Siemens Healthcare, Erlangen, Germany). The MRI protocol included the breath-hold Gradient-Echo (GRE) T1-weighted perfusion sequence (TR = 2.8 ms, TE = 1.12 ms, TI = 110 ms, flip angle = 50°, resolution 2.7 mm x 3.3 mm and slice thickness 8 mm, and ~70 time frames) acquired during gadolinium administration (0.1 mmol/kg of body weight of Gd-DOTA, Dotarem, Guerbet, Paris, France) at a flow rate of 3 ml/s followed by flushes of 20 ml of saline both at rest and after CPT, with an interval of 15 minutes between the two acquisitions to ensure an adequate gadolinium wash-out from the first injection. The field of view (FOV) was oriented according to the cardiac short axis view and large enough (~350 mm) in order to include both the left ventricular myocardium and a large portion of splenic parenchyma in the same images. Three to four slices were acquired per beat, and subjects were requested to breathe shallowly.

### 2.3. Cold Pressor Test

**Figure 1:**
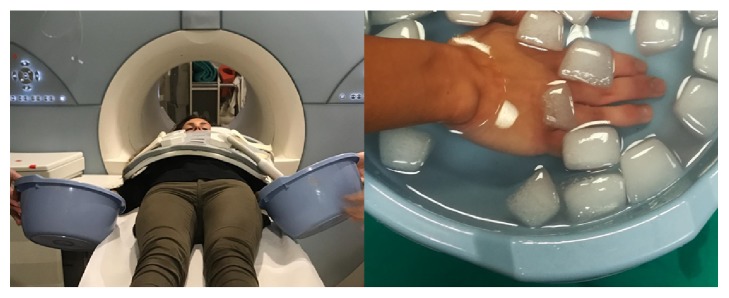
Cold pressor test execution: both hands are completely immersed in ice water for four minutes. Only after the end of the 4 minutes was the perfusion MRI performed.

The CPT was carried out through the complete immersion of both hands in freezing water, for four minutes, as shown in Figure 1.

### 2.4. Image Analysis

GRE T1-weighted images were evaluated using a dedicated software (Cvi42 v5.3.0, Circle Cardiovascular Imaging Inc., Calgary, Alberta, CA). An 8-10 cm^2^ region of interest (ROI) was drawn within the splenic parenchyma in each image and time frame, both at rest and after CPT, while avoiding inclusion of intraparenchymal vascular structures. We also calculated myocardial perfusion at rest and after CPT in order to evaluate myocardial blood flow (MBF): the endocardial and epicardial contours were outlined and copied to all the dynamic images and then manually adapted. Further ROIs were outlined inside the left ventricular cavity to measure the flow of arterial blood pool (ABP).

**Figure 2:**
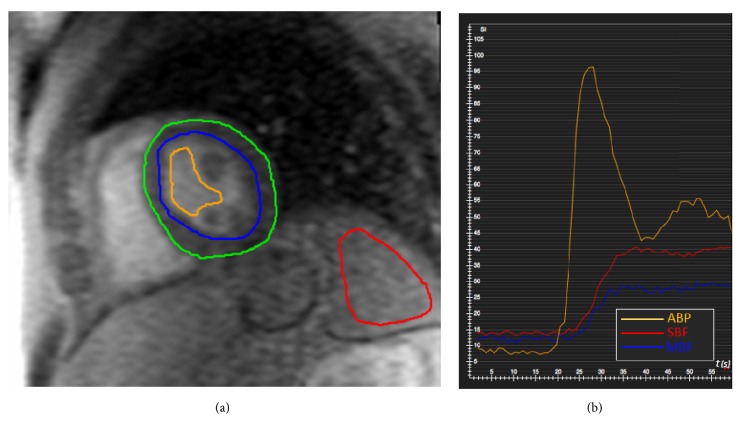
(a) Signal intensity (SI) values were measured in each time frame of the perfusion sequence by drawing 9 cm^2^ regions of interest (ROI) within the spleen parenchyma (red line) to calculate SBF. ROIs within the left ventricle cavity (orange line) were drawn for ABP quantification. To assess MBF, SI of the whole myocardium between epicardial (green) and endocardial (blue) layers was measured. (b) The graph shows the SI-to-Time curves of ABP (orange), SBF (red), and MBF (blue). ABP= arterial blood pool; SBF= splenic blood flow; MBF= myocardial blood flow.

The mean and standard deviation values of signal intensity (SI) were measured for every ROI. Signal-to-time curves were automatically generated for splenic and myocardial perfusion and ABP (Figure 2).

Blood flow parameters (SBF, MBF, and ABP) were calculated by using the deconvolution method [13], converting SI values into concentrations, using the SI equation for the imaging pulse sequence, as previously described [13]. In particular, the amount of contrast agent (CA), represented by the spleen concentration curve Cs(t), is related to the tracer concentration at the inlet (i.e., the input function, represented by the blood concentration curve Cb(t)) convolved with the impulse response function (IRF), as described by the following expression (in the text):

(1) $$\begin{array}{c} C_{s}\left(\;t\;\right)=IRF\left(\;t\;\right)⊗C_{b}\left(\;t\;\right)=SBF⊗\left(\;h\left(\;t\;\right)⊗C_{b}\left(\;t\;\right)\;\right) \end{array}$$

where ⊗ indicates the convolution operator, SBF is the rate of flow, and h(t) is the response function (which represents the fraction of the tracer in the spleen at time t). Hence, SBF is simply determined as SBF = max (h(t)). All results were expressed as mean ± standard deviation. All tests were performed using an in-house script developed on the Matlab software platform (version 7.9.0.529; MathWorks, R2009b), using a deconvolution algorithm that employs the Fermi function as described in a previous paper [13].

### 2.5. Statistical Analysis

The software we used for the statistical analysis was SPSS (version 22.0; SPSS Inc., Chicago, Illinois). Most of the statistical tests were performed using paired comparisons.

A Kolmogorov–Smirnov test was used to assess if the obtained SBF, MBF, and ABP data were normally distributed. These results were tested for significant differences by applying a t-test for independent samples.

SBF, MBF, and ABP values are presented as ml/g/min and cardiac frequencies as beats per minute (BPM). Normally distributed variables are reported as mean and standard error unless otherwise indicated. Bivariate correlations were assessed using Pearson's or Spearman's coefficient. Receiver-operating characteristic (ROC) curve analysis was performed to calculate the optimal cut-off value to distinguish SBF values obtained at rest and after CPT.

P-values (two sided) of less than 0.05 were considered to be statistically significant.

## 3. Results

Every enrolled subject successfully performed the entire MRI study and was able to keep the hands in freezing water for four minutes during sequence acquisitions, without any serious collateral effect.

There were no significant changes in heart rate between the acquisitions at rest and after CPT (71.9 ± 9.5 Vs. 71.5 ± 12.1 bpm, respectively, p=n.s.), whereas the blood pressure values were greater after CPT than at rest (122/72 Vs. 132/82 mmHg, respectively, p<.005 for both diastolic and systolic average blood pressure values). We also observed an increase in Rate Pressure Product (RPP) (8797.5 ± 1392 vs 9463 ± 1707, p<0.05).

**Figure 3:**
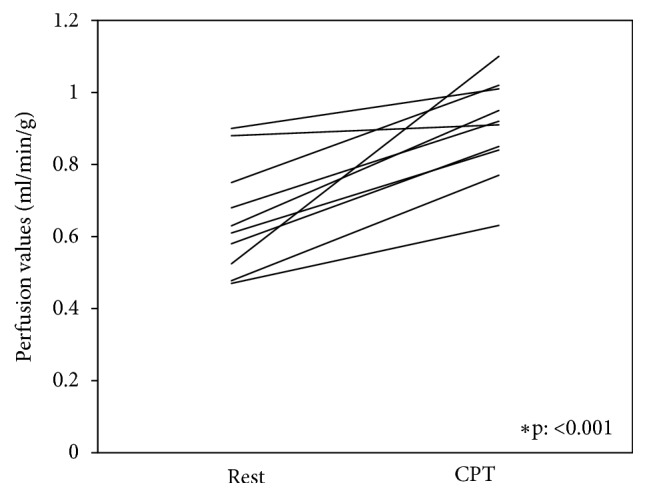
Individual comparison of spleen blood flow (SBF) at rest and during the CPT for each healthy subject.

A significant increase of SBF was observed after CPT (SBF at rest: 0.65 ± 0.15 ml/min/g Vs SBF after CPT: 0.90 ± 0.14 ml/min/g, p: <0.001; Figure 3).

**Figure 4:**
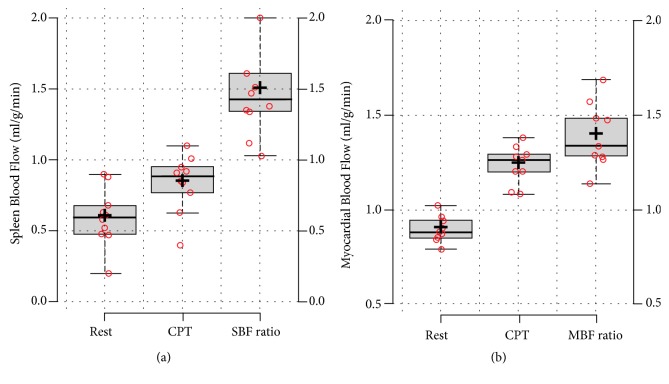
SBF and MBF values at rest and after CPT.

The CPT-induced mean increase of SBF was 43% ± 29%. The coefficient of variance was 23% for the rest examination and 15% for the CPT. A significant increase was also found in MBF (MBF at rest: 0.90 ± 0.068 ml/min/g Vs MBF after CPT: 1.22 ± 0.098 ml/min/g, p<0.005, Figure 4) and ABP (ABP at rest: 66.15 ± 32.1 ml/min/g vs. ABP after CPT: 77.26 ± 41.22 ml/min/g) after applying the CPT (Table 1). Bivariate Correlations analysis showed inverse correlation between SBF and ABP values measured both at rest and after CPT (r [SBFrest Vs. ABPrest] = -.834; r [SBFcpt Vs. ABPcpt] = -.679, p<.05 for both). However, no significant relationships have been found between MBF and SBF/ABP values at rest or after CPT and between SBF, MBF, and ABP changes in response to CPT (r [SBF ratio Vs. MBF ratio] = 0.131 and r [SBF ratio Vs. ABP ratio] = -0.419, p: 0.229-0.719, respectively; graphics available here as supplementary material online). The ROC curve analysis of pooled SBF values showed an area under the curve of 0.851 (95%CI: 0.656-0.988), with an optimal SBF threshold of 0.68 (sensitivity: 80%, specificity: 88.9%, and diagnostic accuracy: 79%) in distinguishing between SBF measured at rest or after CPT.

## 4. Discussion

Our experience confirmed that the CPT is a simple and safe test that did not provoke any negative side effects in any enrolled subjects.

The main result of our study was that the CPT induced an increase in SBF in all cases of our cohort, even though this increase was heterogeneous (43% ± 29%).

The physiological response triggered by the stimulation of cutaneous “cold receptors” activates a complex thermoregulatory system driven by the central nervous system in which the hypothalamus plays a preeminent role [14, 15]. In particular, the rapid decrease of hand temperature induced by the 4-minute immersion in ice water provokes the release of NE in bloodstream by the sympathetic nervous system. This stimulates a number of different responses such as the increase in heart rate, peripheral vasoconstriction, tensing of muscles, and higher metabolic rate [14, 15].

This neurotransmitter elicits its vasomotion effects by binding to the three types of ARs located on the surface of vascular smooth muscle cells: *α*1, *α*2, and *β*2. *β*2 adrenoceptors are involved solely in vasodilation (they are predominant in heart and lung arteries [16]), whereas *α*1- and *α*2-ARs are responsible for vasoconstriction, and they are predominantly present in smaller blood vessels or arterioles, especially in skin, muscle, and renal and mesenteric arteries [16].

In the human spleen, NE controls red pulp smooth muscle cells by binding predominantly to *α*-ARs and provoking vasoconstriction [3]. The source of this NE was proposed to be sympathetic neurons originating in the celiac ganglia and projecting into the spleen, even though the effect of circulating catecholamines secreted by the adrenal glands is well documented [15].

Furthermore, other studies demonstrated that the human spleen is capable of contracting and regulating its volume, as a consequence of humoral stimulation mediated via ARs (*α*1, *α*2, *β*1, and *β*2) located in the splenic capsule and parenchyma [2, 17]. In particular, Bakovic et al. [1, 18] showed that the infusion of low doses of epinephrine could induce the spleen capsule to contract and the spleen volume to decrease. How the volumetric modifications of the spleen and its contraction result in a variation of the SBF is still unknown. However, some studies have demonstrated an inverse correlation between splenic perfusion and spleen size in patients with haematological disorders [19] and hepatic cirrhosis [20].

In a systemic perspective, the NE secretion caused by the cold stimulus decreases vascular conductance of skin and of brachial, celiac, superior mesenteric, and renal arteries [21–25]. This provokes an increase in the mean circulatory filling pressure (PMFC) [26], which was described by Guyton as “the entire circulatory system if the heart were stopped suddenly and the blood were redistributed instantaneously in such a manner that all pressures were equal” [27, 28].

Nevertheless, a comprehensive explanation of the role of the spleen in the regulation of both blood pressure and blood volume after physical and physiological stress still remains to be revealed [29, 30].

According to our results, we can assume that the combination of these complex mechanisms occurring immediately after the cold stimulus (vasomotion, redistribution of blood volume from the periphery to those vascular districts with lower resistance, and spleen contraction and volume reduction) results in an increase of SBF. Further studies investigating the combination of the effect of the adrenergic stimulus on splenic contraction by measuring spleen volume and vasomotility could clarify these complex, integrated mechanisms.

Furthermore, in our study, also the MBF increased after CPT in healthy subjects (36.5% ± 17%), changes that were not statistically associated to the SBF modifications.

The MBF changes after CPT have been investigated in several studies, which established that the cold stimulus induces coronary vasodilation in normal subjects with preserved endothelial function [11, 31]. Indeed, sympathetic activation increases the cardiac oxygen demand, activating nitric oxide (NO) production by endothelial cells of the intima and causing coronary vasodilation [31, 32].

Systemic disorders affecting the endothelial function, such as those seen in smokers [10], in patients with early atherosclerosis [33], or in patients with diabetes [12], alter these mechanisms and hamper the production of NO, resulting in an impairment of vasodilation response [34–36].

Moreover, some pathological conditions may be associated with dysregulation of microvascular function evoked by cold, as reported by Quarta et al. [37], which demonstrated a paradoxical cold-induced vasoconstrictor effect of sympathetic activation on coronary microcirculation in patients affected by systemic sclerosis leading to transient myocardial ischemia. The authors defined this vasomotility dysfunction as “cardiac Raynaud phenomenon”.

To our knowledge, there are no previous published studies that evaluate vascular splenic response to hypothermia using a direct quantification of blood flow. Further studies are desirable to explore the complex interaction between all different mechanisms involved in the splenic vasomodulation activated by cold.

The main limitation of our study is the relatively small sample size that could have resulted in wide variance of the data; however, the study population was large enough to obtain statistically significant results. We did not measure NE blood levels or spleen volume that could monitor the activity of sympathetic nervous system and provide further information for the data interpretation, in particular regarding the role of NE-mediated SBF-modulation.

We did not have any reference test to verify that all subject received an adequate cold stimulation. Therefore, it is possible that some data could be hampered by a no- or suboptimal response from ineffective hypothermic stress, which does not seem to have substantially affected the overall results. The lack of a reference method to verify the effectiveness of the CPT, in particular, impedes calculating a SBF threshold value that could be used as adequacy indicator of CPT success.

Finally, although the test was substantially well-tolerated by all subjects enrolled and it did not provoke any systematic disorders or injuries, the CPT caused discomfort and an unpleasant experience for the most volunteers. Therefore, we cannot exclude that for some patients the cold stimulus might be very painful and intolerable.

## 5. Conclusions

The CPT provokes an increase in SBF in healthy subjects, as result of the sympathetic activation on splenic vessels, spleen contraction, volume modulation, and centralization of blood circulation. The increase in SBF could be used as marker of adequacy of hypothermic stimulus after CPT.

## Figures and Tables

**Table 1:** Main population parameters concerning age, gender, Body Mass Index, systolic and diastolic blood pressure, heart beat, Rate Pressure Product, splenic blood flow (SBF), arterial blood pool (ABP), and myocardial blood flow (MBF). All the results are presented at rest and after CPT.

Population Parameters			
Age (mean ± SD)	43.7 ± 7.3		
Gender (M, %)	5 (50%)		
BMI (mean ± SD)	23.3 ± 1.97		

	Before CPT	After CPT	p

Systolic blood pressure (mmHg, mean ± SD)	122 ± 7.53	132 ± 5.38	<0.005
Diastolic blood pressure (mmHg, mean ± SD)	72 ± 7.89	82 ± 5.87	<0.005
Heart Beat (beat per minute, mean ± SD)	71.9 ± 9.5	71.5 ± 12.1	n.s.
Rate Pressure Product (mean ± SD)	8797.5 ± 1392	9463 ± 1707	<0.05
SBF (ml/min/g, mean ± SD)	0.65 ± 0.15	0.90 ± 0.14	<0.001
ABP (ml/min/g, mean ± SD)	66.15 ± 32.1	77.26 ± 41.22	n.s.
MBF (ml/min/g, mean ± SD)	0.90 ± 0.068	1.22 ± 0.098	<0.001

## Data Availability

The database with clinical, SBF, MBF, and ABP values used to support the findings of this study is available as supplementary information file.

## Supplementary Materials

Supplementary Materials Supplementary Figure: Bivariate correlation between MBF, ABP, and SBF at rest and after CPT and MBF vs. SBF increase (%) after CPT using Pearson's or Spearman's coefficient.
