# Supplementary material for: Splenic Blood Flow Increases after Hypothermic Stimulus (Cold Pressor Test): A Perfusion Magnetic Resonance Study
Source: Biomed Res Int. 2019 May 23;2019:8437927. doi: 10.1155/2019/8437927 (PMC6556242; doi:10.1155/2019/8437927)
Supplement: Supplementary Materials — Supplementary Figure: Bivariate correlation between MBF, ABP, and SBF at rest and after CPT and MBF vs. SBF increase (%) after CPT using Pearson's or Spearman's coefficient. [file 8437927.f1.pdf]

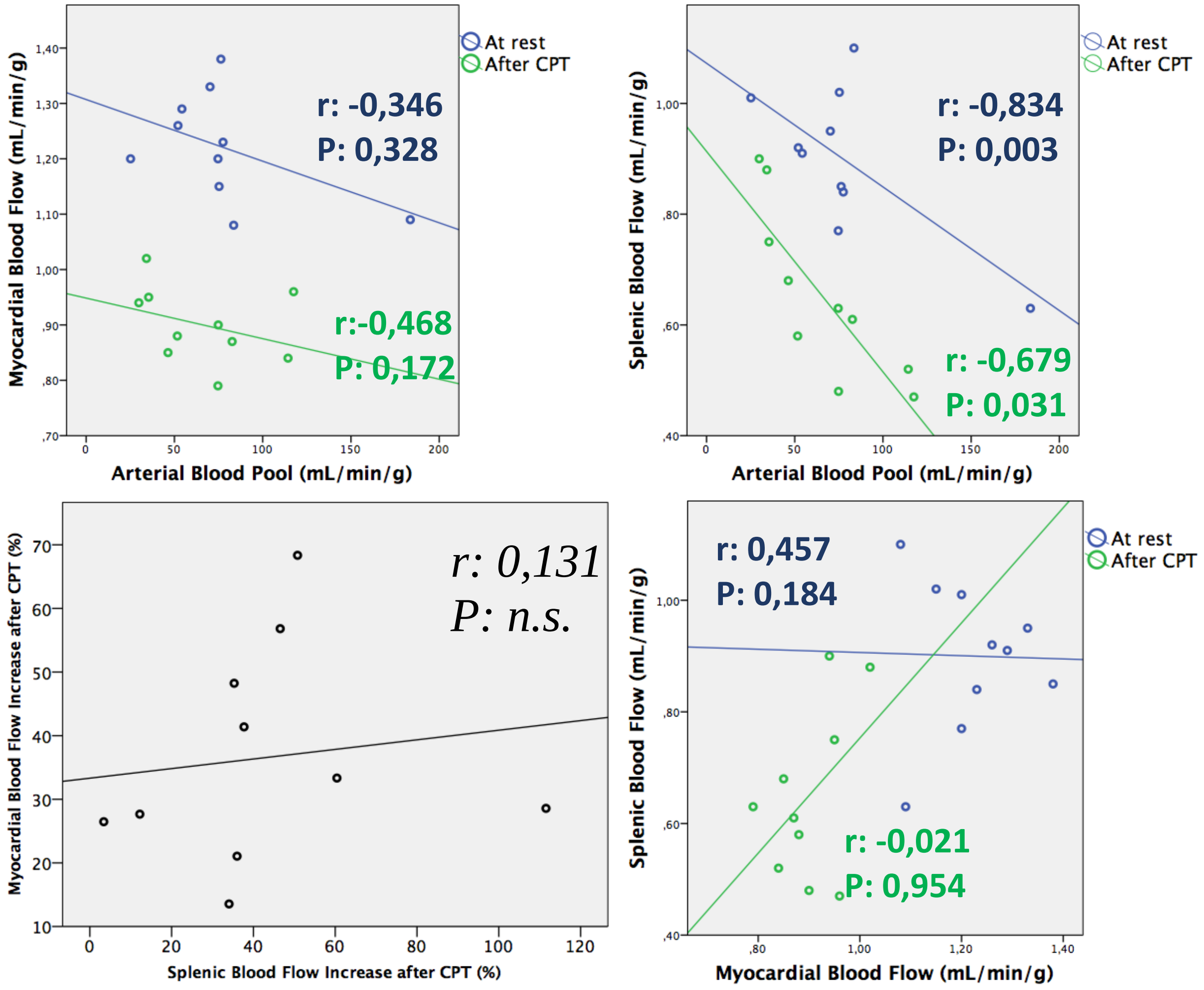

Supplementary Figure: Bivariate correlation between MBF, ABP, and SBF at rest and after CPT and MBF vs. SBF increase (%) after CPT using Pearson's or Spearman's coefficient.
